# Geospatial Variation and Determinants of Time to Pregnancy Loss Among Reproductive‐Aged Women in East Africa: Bayesian Spatial Frailty Model

**DOI:** 10.1155/bmri/4637028

**Published:** 2026-06-19

**Authors:** Solomon Keflie Assefa, Meron Asmamaw Alemayehu, Tadesse Awoke Ayele

**Affiliations:** ^1^ Department of Epidemiology and Biostatistics, Institute of Public Health, College of Medicine and Health Sciences, University of Gondar, Gondar, Ethiopia, uog.edu.et; ^2^ Department of Public Health, Pawi Health Science College, Pawi, Ethiopia

**Keywords:** Bayesian spatial, Bayesian survival, East Africa, frailty, pregnancy loss, spatial survival

## Abstract

**Background:**

Pregnancy loss is the terminated pregnancy before the completed pregnancy time. Spatial location can significantly influence pregnancy outcomes, yet geographic disparities in the risk of pregnancy loss remain poorly studied in East Africa. This study is aimed at examining the spatial variation and determinants of time to pregnancy loss among women of reproductive age in the region.

**Methods:**

A Bayesian spatial survival model with an intrinsic conditional autoregressive approach was utilized to identify factors related to time to pregnancy loss across 169 regions in 9 East African countries using secondary data from recent demographic and health surveys (2015–2023). A spike‐and‐slab prior was used for variable selection. Model comparison utilized deviance information criteria, Watanabe–Akaike information criterion, and log pseudo marginal likelihood, with diagnostics from Cox and Snell residuals.

**Results:**

Pregnancy loss showed significant spatial clustering. Earlier occurrence was observed among women aged 25–34 years (*ϕ* = 0.712; 95% CrI: 0.680–0.744) and ≥ 35 years (*ϕ* = 0.326; 95% CrI: 0.307–0.344), those living with their husbands (*ϕ* = 0.893; 95% CrI: 0.856–0.932), with primary education (*ϕ* = 0.939; 95% CrI: 0.892–0.987), regular media exposure (*ϕ* = 0.854; 95% CrI: 0.819–0.889), employed (*ϕ* = 0.818; 95% CrI: 0.788–0.850), and attending antenatal care (*ϕ* = 0.964; 95% CrI: 0.933–0.996). In contrast, college‐educated women (*ϕ* = 1.109; 95% CrI: 1.024–1.203), rural residents (*ϕ* = 1.144; 95% CrI: 1.102–1.190), low parity (*ϕ* = 14.93; 95% CrI: 13.853–16.132), and grand multiparity (*ϕ* = 22.088; 95% CrI: 20.131–24.247) were associated with longer time to pregnancy loss. After adjusting for individual‐level factors, residual variation in the hazard of pregnancy loss remained.

**Conclusions:**

Pregnancy loss remains a significant public health challenge in East Africa, with significant geographic variation. Spatial analysis can guide region‐specific healthcare strategies and targeted interventions.

## 1. Introduction

Better maternal health and pregnancy outcomes are significant public health priorities [1]. The definition of pregnancy loss varies significantly, with countries using different criteria and standards for reporting [2–5]. Pregnancy loss is a collective term used to describe pregnancies that failed to produce a live birth [6]. The International Classification of Diseases (ICD‐10) defines fetal death as the death of a fetus before complete expulsion or extraction, regardless of pregnancy duration [2]. Early pregnancy loss (miscarriage and abortion) and stillbirth (late pregnancy loss) are terms used to describe fetal loss at different stages of pregnancy.

Pregnancy loss is a time‐sensitive event. Each year, approximately 23 million losses occur before 12 weeks of gestation [7], whereas 2.6 million stillbirths (≥ 28 weeks) are reported, with both outcomes disproportionately affecting sub‐Saharan Africa [8–10]. This temporal divide reflects distinct etiologies from chromosomal anomalies in early pregnancy [11, 12] to systemic healthcare gaps in later stages [13, 14], yet most studies fail to account for timing‐dependent risks. Understanding when losses occur is essential to unraveling distinct risk factors and designing stage‐specific interventions.

Risk factors for pregnancy loss vary significantly by gestational timing. Early losses are predominantly attributed to chromosomal abnormalities [11, 12, 15], whereas later losses correlate with factors such as maternal age extremes [13, 16], multiple previous births [17], inadequate antenatal care (ANC), and obstetric complications [18, 19] The timing of pregnancy loss critically impacts women′s health; whereas early losses often have minimal consequences, later losses significantly increase risks of physical complications, emotional trauma, and future pregnancy challenges, demanding comprehensive care [20–22].

Spatial variation in pregnancy loss is evident at the global level and is strongly influenced by the socioeconomic status of regions. Both early and late pregnancy losses remain high in sub‐Saharan Africa [23, 24]. The region′s share of global stillbirths has increased from 27% in 2000 to 42% in 2019 [8]. Although developed countries have made significant progress in reducing stillbirths, East Africa continues to lag behind and struggles to meet ENAP targets [25]. If current trends persist, projections indicate that by 2030, over half of all global stillbirths will occur in sub‐Saharan Africa, predominantly in East Africa [8].

Despite the high burden of pregnancy loss and the importance of geographic variation for targeted interventions, no studies in Africa have jointly examined spatial patterns and the timing of pregnancy loss. Existing research primarily relies on conventional regression models [26–28], which fail to account for spatial dependence, censoring, or follow‐up time [29, 30].

To address these gaps, Bayesian spatial survival models offer a robust analytical framework for geographically indexed time‐to‐event data [30]. These models incorporate conditional autoregressive (CAR) priors to explicitly model spatial dependence while simultaneously examining predictors of time‐to‐pregnancy loss [29]. Crucially, unlike standard spatial generalized linear models, Bayesian spatial survival approaches can accommodate censored observations [30, 31].

Thus, this study is aimed at investigating the spatial variation and determinants of time to pregnancy loss across East Africa using a Bayesian spatial survival model. By doing so, this study provides critical evidence to inform region‐specific healthcare planning and targeted interventions.

## 2. Methods and Materials

### 2.1. Data Source, Study Period and Setting

The study used recent standardized DHS data (2015–2023) from the official DHS program database (https://dhsprogram.com) for East African countries, including Burundi, Ethiopia, Kenya, Malawi, Mozambique, Rwanda, Tanzania, Zambia, and Zimbabwe [32]. Polygon shapefiles for these regions were obtained from the GADM database (https://gadm.org/).

### 2.2. Study Design and Study Population

**Figure 1:**
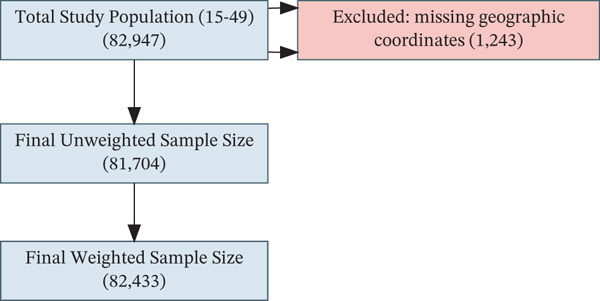
Illustration of sampling procedure.

The study utilized a cross‐sectional design with nationally representative data using multistage stratified cluster sampling. Our study included women aged 15–49 with recent pregnancies, excluding those with missing outcome data or geographic coordinates. Uganda and Madagascar were excluded due to mismatches between DHS regions and shapefile boundaries. The complete sampling approach is illustrated in Figure 1.

### 2.3. Variables and Operational Definition

The study examines the time to pregnancy loss (coded as 1 = Loss, 0 = Censored) among women over the past 5 years. Independent variables include sociodemographic factors, health‐related factors, and community‐level factors.

#### 2.3.1. Pregnancy Loss

Pregnancy loss is defined as the termination of pregnancy irrespective of the duration of gestational age [3, 4]. The event is defined as the occurrence of pregnancy loss within the 5 years preceding the survey, whereas censored cases represent pregnancies without loss in that period. Survival time refers to the gestational duration (in months) from conception to pregnancy loss.

#### 2.3.2. ANC Utilization

ANC utilization is defined as women who attended a minimum of four prenatal care appointments [33].

### 2.4. Data Management and Statistical Analysis

Missing data were first examined using graphical methods and visual plots. Variables missing by design (husband′s education, paternal age, and husband′s occupation for single mothers) were excluded from the final model due to their impact on model parameters and estimation stability. For other variables, Little′s MCAR test indicated they were not missing completely at random, so we assumed missing at random (MAR) and used multivariate imputation by chained equations (MICE) with logistic regression in R (*mice* package) to generate 10 imputed datasets [34]. The imputation model included variables such as cesarean section, ANC, health insurance, and smoking status.

### 2.5. Bayesian Modeling Approach for Spatial Survival Analysis

Spatial correlation in survival outcomes often arises from unmeasured regional similarities. Frailty models are one of the approaches to effectively account for these spatial dependencies while adjusting for known covariate effects [30]. Although Cox PH models are widely used to model time to event outcomes, AFT models offer a simpler interpretation via log‐linear parameters and greater robustness to omitted covariates compared with PH models [35, 36].

The spatial structure can be incorporated into the AFT model by adding a random effect [37];

yij=logtij=xijTβ+vi+ϵij

where *y*_*i* *j*_ is the log of survival time, **x**_*i* *j*_ is the vector of covariates, *β* is the regression coefficient vector, *v*_*i*_ represents the spatial random effect for region *i*, and *ϵ*_*i* *j*_ is the error term.

The AFT model with frailty has survival and density functions [38],

Sxijt=S0expxij⊤β+vijt, fxijt=expxij⊤β+vijf0expxij⊤β+vijt

Where $\beta ={\left(\beta_{1},\cdots ,\beta_{p}\right)}^{⊤}$i s a vector of regression coefficients, *v*_*i*_ is an unobserved frailty associated with *s*_*i*_, and “*S*_0_ (.)” is the baseline survival with density  ^"^ *f*_0_(.)^"^ corresponding to  ^"^ *X*_*i* *j*_ = 0^"^ and  ^"^ *v*_*i*_ = 0^"^.

In the case of spatial survival data, the frailty model can be extended by including a spatial effect as follows:

ηi=xiβ+γi, γi=vi+wi,

where the frailty term *γ*_*i*_ incorporates the effects of both heterogeneity (via the nonspatial frailty *v*_*i*_) and spatial dependence (through the spatial frailty *w*_*i*_) [30].

In modeling time to pregnancy loss across nine East African countries, we incorporated region‐level frailty using an intrinsic conditional autoregressive (ICAR) model. The model fitting was conducted using the *spBayesSurv* package in R [39].

### 2.6. Prior Specification

Given the lack of previous applications of spatial frailty models to pregnancy loss and the absence of prior information on spatial random effects or covariate effects, we employed vague priors, specifying regression coefficients as follows:

β∼N01000,

For the baseline hazard function scale parameter (S0), we assigned Bayesian nonparametric vague priors to the shape (*α*) and scale (*θ*) parameters of the two‐parameter Burr Type XII transformed Bernstein polynomial (TBPL) distribution as [40, 41],

S0·α,θ∼TBPL α,Sθ ·,α∼Γ 0.01,0.01 and θ∼N2 01000,

For the spatial frailty terms *V*_*i*_, vague priors was assigned to the precision parameter *τ* in the ICAR model with a gamma distribution [42],

v1,⋯,vmTτ∼ICAR τ2,τ−2∼Γ0.01,0.01

These noninformative priors allow the data to strongly influence the posterior distribution.

Variable selection was performed using a spike‐and‐slab prior with a Bernoulli distribution via the *spBayesSurv* R package [39, 43]. Model diagnostics were conducted using Cox–Snell residuals and model fit was assessed with the deviance information criterion (DIC), log pseudo marginal likelihood, and Watanabe–Akaike information criterion (WAIC) [39, 44]. Detailed model description and prior specifications are provided in Annex S1.

### 2.7. Ethical Consideration and Clearance

This study used secondary data from the Demographic and Health Surveys (DHS) program (https://www.dhsprogram.com) by clearly specifying the research purpose. The data were accessed on June 24, 2024. To protect spatial confidentiality, the DHS randomly displaces GPS coordinates of survey respondents, shifting urban clusters by 0–2 km and rural clusters by 0–5 km, with 1% of rural clusters displaced by up to 10 km. Further ethical considerations can be found on the DHS website.

## 3. Results

### 3.1. Descriptive Characteristics of Study Participants

The study comprised 82,433 women across nine East African countries, of whom 12,237 (14.8%) experienced pregnancy loss. Sociodemographic data showed 60,909 women (73.9%) resided rurally, 67,368 (81.7%) cohabited with partners, and 39,245 (47.6%) completed primary education. Socioeconomic indicators revealed 33,927 women (41.2%) were in the poorest wealth quintile, whereas 31,222 (37.9%) reported no media exposure. Reproductive health characteristics included 21,096 grand multiparous women (25.6%), 37,942 modern contraceptive users (46.0%), and 30,921 (44.7%) with no ANC visits. Health service utilization showed 5764 cesarean deliveries (8.4%) and 57,426 women (86.1%) without health insurance. Only 531 participants (0.7%) reported tobacco use (Table 1).

**Table 1:** Women′s baseline characteristics by loss of pregnancy of women in East Africa using recent DHS (2015–2023).

Variable	Categories	Weighted frequency (%)	No. of pregnancies without loss (%)	No. of pregnancy loss (%)
Countries	Burundi	9210 (11.17)	8084 (9.81)	1126 (1.37)
	Ethiopia	7654 (9.29)	7066 (8.57)	588 (0.71)
	Kenya	15,733 (19.09)	11,737 (14.24)	3996 (4.85)
	Malawi	13,930 (16.9)	12,883 (15.63)	1047 (1.27)
	Mozambique	7813 (9.48)	6488 (7.87)	1325 (1.61)
	Rwanda	6590 (7.99)	5730 (6.95)	860 (1.04)
	Tanzania	8865 (10.75)	6713 (8.14)	2152 (2.61)
	Zambia	7401 (8.98)	6852 (8.31)	549(0.67)
	Zimbabwe	5236 (6.35)	4644 (5.63)	592 (0.72)

Maternal age	15–24	23,924 (29.02)	21,574 (26.17)	2350 (2.85)
	25–34	36,768 (44.6)	32,119 (38.96)	4649 (5.64)
	35 and above	21,740 (26.32)	16,502 (20.02)	5238 (6.35)

Place of Residence	Urban	21,524 (26.11)	17,146 (20.8)	4378 (5.31)
	Rural	60,909 (73.89)	53,050 (64.36)	7859 (9.53)
Maternal occupation	Not working	29,022 (35.22)	25,616 (31.08)	3406 (4.13)
	Working	53,385 (64.78)	44,555 (54.07)	8830 (10.72)

Women educational level	No education	17,443 (21.16)	15,392 (18.67)	2051 (2.49)
	Primary	39,245 (47.61)	33,491 (40.63)	5754 (6.98)
	Secondary	20,878 (25.33)	17,631 (21.39)	3247 (3.94)
	College and above	4867 (5.9)	3682 (4.47)	1185 (1.44)

Marital status	Not living with husbands	15,065 (18.28)	12,794 (15.52)	2271 (2.75)
	Living with husbands	67,368 (81.72)	57,402 (69.63)	9966 (12.09)

Wealth status	Poor	33,927(41.16)	29,917 (36.29)	4010 (4.86)
	Middle	15,733 (19.09)	13,585 (16.48)	2148 (2.61)
	Rich	32,773 (39.76)	26,693 (32.38)	6080 (7.37)

Media exposure	No media	31,222 (37.88)	28,002 (33.97)	3220 (3.91)
	Has media	51,211 (62.12)	42,194 (51.19)	9017 (10.94)

Paternal age (*n* = 67,368)	< 25	7921 (11.76)	7096 (10.53)	825 (1.22)
	25–45	49,880 (74.04)	43,182 (64.1)	6698 (9.94)
	45–95	9567 (14.2)	7124 (10.57)	2443 (3.63)

Husband education Level (*n* = 67,368)	No education	13,223 (19.63)	11,777 (17.48)	1446.52 (2.15)
	Primary	30,250 (44.9)	25,819 (38.33)	4431 (6.58)
	Secondary	18,352 (27.24)	15,536 (23.06)	2815 (4.18)
	College and above	5543 (8.23)	4269 (6.34)	1274 (1.89)

Husband occupation (*n* = 67,351)	Not working	6839(10.15)	6108 (9.07)	N(1.08)
	Working	60,512 (89.85)	51,280 (76.14)	9232 (13.71)
Smoking habit (*n* = 74,892)	No	74,361 (99.29)	64,067 (85.55)	10,294 (13.75)
	Yes	531 (0.71)	437 (0.58)	94 | (0.12)
Delivered by cesarean section (*n* = 69,059)	No	63,295 (91.65)	58,511 (84.73)	4,784 (6.93)
	Yes	5764 (8.35)	5008 (7.25)	756 (1.09)

Parity	Nullipara	1249 (1.52)	—	1249 (1.52)
	Low multipara	60,088 (72.89)	52,013 (63.1)	8075 (9.8)
	Grand multipara	21,096 (25.59)	18,183 (22.06)	2913 (3.53)

ANC visit (*n* = 69,156)	No visit	30,921 (44.71)	28,862 (41.73)	2059 (2.98)
	Has visit	38,236 (55.29)	34,750 (50.25)	3481 (5.04)
Covered by health insurance (*n* = 66,700)	No	57,426 (86.1)	50,566 (75.81)	6860 (10.28)
	Yes	9273 (13.9)	7892 (11.83)	1381 (2.07)
Preceding birth interval (*n* = 62,245)	Less than 2 years	9834 (15.8)	8524 (13.69)	1310 (2.11)
	2 years and above	52,411 (84.2)	44,958 (72.23)	7452 (11.97)

Contraceptive	Nonusers	42,004 (50.96)	34,804 (42.22)	7201 (8.74)
	Traditional	2487 (3.02)	1916 (2.32)	572 (0.69)
	Modern	37,942 (46.03)	33,477 (40.61)	4465 (5.42)

Total		**82,433**	**70,196 (85.16)**	**12,237 (14.84)**

*Note:* Husband′s education, paternal age, and husband’s occupation were missing by design for cases where women were single. The total sample size is indicated in brackets for variables with missing data.

### 3.2. Model Diagnostics and Comparison

Prior to implementing the spatial survival model, a standard survival analysis was conducted. The Schoenfeld residual test indicated a violation of the proportional hazard assumption. Consequently, the analysis was extended to a spatial survival framework using an ICAR frailty within a semiparametric accelerated failure time (AFT) model. The AFT model with ICAR frailty and a log‐normal distribution provided the best fit, with the lowest DIC and WAIC and the highest LPML (Table 2). A Bayes factor of 5209.56 further confirmed strong evidence favoring the semiparametric ICAR model over the parametric alternative. The Cox–Snell residual plots showed an overall good model fit. Detailed modeling procedures and diagnostics are presented in Annex S2.

**Table 2:** Model comparisons with and without frailty for different baseline distributions.

Distribution	No frailty			ICAR frailty		
	DIC	LPML	WAIC	DIC	LPML	WAIC
Lognormal	150,417.5	−73,211.76	150,521.5	113,905.1	−56,952.89	113,905.8
Weibull	169,841.1	−78,253.70	169,901.2	125,921.6	−62253.70	125,934.2
Log logistic	178,459.9	−84,496.92	178,541.9	128,989.3	−64496.92	128,993.9

Abbreviations: DIC, deviance information criterion; LPML, log pseudo marginal likelihood; WAIC, Watanabe–Akaike information criterion.

### 3.3. Determinants of Time to Pregnancy Loss in East Africa

In the multivariable Bayesian spatial survival analysis, several factors were significantly associated with the timing of pregnancy loss (Table 3). Compared with women aged 15–24 years, those aged 25–34 years had an acceleration factor (*ϕ*) of 0.712 (95% CrI: 0.680–0.744), whereas women aged 35 years and above had a *ϕ* of 0.326 (95% CrI: 0.307–0.344). These results suggest that, after adjusting for other covariates and spatial frailty, pregnancy loss occurs approximately 28.8% earlier among women aged 25–34 years and 67.4% earlier among those aged 35 years and above, relative to younger women.

**Table 3:** Posterior inference of regression coefficients for lognormal AFT model with ICAR frailty.

Variables	Categories	Estimate	Acceleration factor (*Φ*)	L‐95% CrI	U‐95% CrI
		Mean			
Women′s age	15–24	1	1		
	25–34	−0.340	0.712	0.680	0.744
	> 35	−1.122	0.326	0.307	0.344

Residence	Urban	1	1		
	Rural	0.135	1.144	1.102	1.190
Marital status	Not living together	1			
	Living together	−0.113	0.893	0.856	0.932

Women educational level	No education	1	1		
	Primary school	−0.063	0.939	0.892	0.987
	Secondary school	0.052	1.053	0.991	1.117
	College and above	0.104	1.109	1.024	1.203

Covered by health insurance	No	1	1		
	Yes	−0.025	0.975	0.926	1.029
Media exposure	No	1	1		
	Yes	−0.158	0.854	0.819	0.889
ANC	No	1	1		
	Yes	−0.037	0.964	0.933	0.996
Maternal occupation	Not working	1	1		
	Working	−0.201	0.818	0.788	0.850

Parity	Nullipara	1	1		
	Low multipara	2.704	14.932	13.853	16.132
	Grand multipara	3.095	22.088	20.131	24.247


Women living with their husbands had a shorter time to pregnancy loss (*ϕ* = 0.893; 95% CrI: 0.856–0.932), indicating a 10.7% faster occurrence of pregnancy loss compared with those not living with their husbands. Similarly, women with primary education experienced pregnancy loss 6.1% earlier (*ϕ* = 0.939; 95% CrI: 0.892–0.987) than women with no formal education. In contrast, women with college‐level education or higher had longer survival times to pregnancy loss (*ϕ* = 1.109; 95% CrI: 1.024–1.203).

Women with regular media exposure had a shorter survival time (*ϕ* = 0.854; 95% CrI: 0.819–0.889), experiencing pregnancy loss 14.6% earlier than those without regular media exposure. Likewise, employed women had a higher risk of earlier pregnancy loss (*ϕ* = 0.818; 95% CrI: 0.788–0.850), corresponding to an 18.2% shorter time to the event compared with unemployed women. Attendance at ANC was also associated with a slightly shorter time to pregnancy loss (*ϕ* = 0.964; 95% CrI: 0.933–0.996), indicating a 3.7% faster occurrence relative to women who did not attend ANC. Regarding parity, women with low parity had a *ϕ* of 14.93 (95% CrI: 13.853–16.132), and those with grand multiparity had an even higher *ϕ* of 22.088 (95% CrI: 20.131–24.247), both relative to nulliparous women. Finally, rural residence was associated with a longer survival time to pregnancy loss compared with urban residence (*ϕ* = 1.144; 95% CrI: 1.102–1.190), suggesting that, after controlling for other factors and spatial frailty, pregnancy loss tends to occur later among rural women than among their urban counterparts.

### 3.4. Unobserved Heterogeneity in Pregnancy Loss Risk

The Bayesian spatial frailty model, adjusted for known risk factors, revealed significant unobserved heterogeneity in pregnancy loss risk. The posterior estimate of the conditional CAR frailty variance was 0.288 [95% CrI: 0.218–0.371], with a median of 0.341. This suggests substantial variability in risk beyond observed factors included in the model.

**Figure 2:**
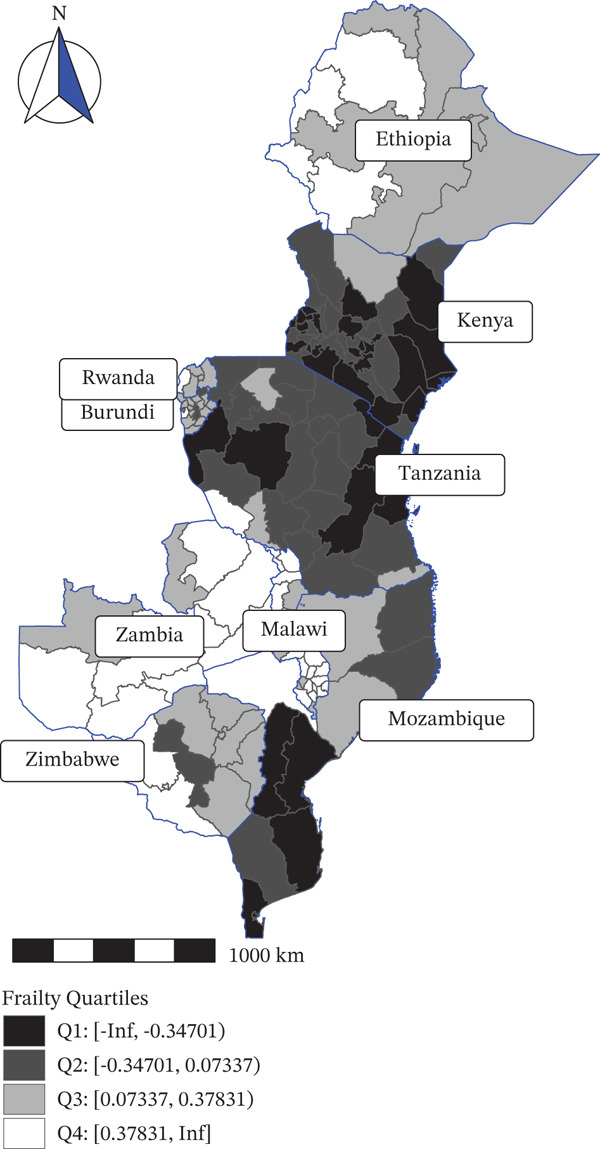
Posterior mean frailties in East Africa regions.

To illustrate spatial effects, we present the mean of the posterior spatial random effects categorized into quartiles. In the AFT spatial model, frailties are additive to the logarithm of survival times, so regions with lower frailties are expected to have shorter survival times. The map displayed in Figure 2 shows 169 regions of East Africa. Regions with darker colors, which indicate lower spatial random effects (and thus shorter survival times), are primarily found in southern Mozambique, eastern Kenya, and both eastern and western Tanzania (Figure 2).

## 4. Discussion

This study examined spatial variation and determinants of time to pregnancy loss across nine East African countries using recent DHS data and a Bayesian spatial frailty model. Key individual‐level factors were identified, and the model effectively accounted for unobserved regional heterogeneity. Findings revealed significant geographic differences in the timing of pregnancy loss among reproductive‐aged women.

In this study, several factors were significantly associated with the timing of pregnancy loss after adjusting for other covariates and accounting for spatial frailty. Being in age 25–34 accelerated pregnancy loss by 28.8% and over 35 by 67.4% as compared with those aged 15–24. This finding aligns with previous research indicating a correlation between older maternal age and increased risk of pregnancy loss [45, 46]. This might be due to declining egg quality, increased risk of chronic health conditions, and impaired uterine and placental function with age [47–49].

Women living with their husbands had shorter survival time as compared with those not living with their husbands. This finding is in contrast with a study conducted in North America and Canada [7, 50], likely reflecting contextual differences. In East Africa, marital cohabitation is associated with increased reproductive pressure, shorter birth intervals, limited male involvement in maternal care, and reduced decision‐making autonomy for women [51, 52]. These factors can elevate the risk of pregnancy loss. In contrast, in high‐income settings, living with a partner often implies greater emotional, social, and financial support, which contributes to improved pregnancy outcomes [53, 54].

In this study, regular media exposure was associated with a 14.6% faster occurrence of pregnancy loss. This counterintuitive finding may reflect that women with greater media access are more informed and therefore more likely to recognize and report pregnancy complications early, leading to higher detection rates rather than higher true incidence [55, 56]. In addition, frequent media exposure may be correlated with urban residence, employment, or lifestyle factors such as delayed childbearing and stress, which are known to increase the risk of pregnancy loss [57]. Further research is warranted to disentangle these behavioral and contextual pathways and to better understand the role of media exposure and occupational factors in influencing pregnancy outcomes in East Africa.

In this study, employed women experienced pregnancy loss 18.2% earlier than unemployed women. This association may be explained by occupational and psychosocial factors such as physical strain, long working hours, job‐related stress, and limited rest during pregnancy, which can increase the risk of adverse pregnancy outcomes [58, 59]. In many parts of East Africa, employed women, especially those in informal or labor‐intensive jobs, may also face inadequate workplace protections and limited flexibility to attend antenatal visits or take maternity leave [60]. Furthermore, employment may coincide with urban residence or delayed childbearing, both of which are associated with higher risk of pregnancy loss [60, 61]. These findings highlight the need for improved workplace policies and maternal health interventions that protect pregnant women from occupational hazards.

Maternal education was another significant determinant of the timing of pregnancy loss. Mothers with a college education or higher experienced pregnancy loss 10.9% slower as compared with no education. These findings are in line with previous literature that showed higher education levels often correlate with better socioeconomic status [62], which can further contribute to healthier pregnancies and reduced risk of loss [63, 64]. This could be due to better access to healthcare, improved health practices, and greater awareness of pregnancy‐related risks with education [65, 66]. This implies that enhancing educational opportunities for women could be a crucial strategy for improving pregnancy outcomes.

In this study, women who attended ANC had a shorter survival time. This finding is in contrast with studies conducted in Eastern Ethiopia and Nigeria [67, 68]. This discrepancy could also be due to women with high‐risk pregnancies attending ANC more frequently due to complex health issues [69]. Additionally, late initiation of care and disparities in service quality may also contribute to these findings. A more detailed analysis of ANC timing, service quality, and related factors is needed to better understand these dynamics [70, 71].

In this study, parous women had longer survival times compared with nulliparous women. This finding contrasts with a study conducted in rural Ethiopia [28]. This difference may stem from parous women having more stable reproductive health, whereas nulliparous women could face higher biological or psychological risks [72].

Residence also influenced survival times, with rural women experiencing longer survival times before pregnancy loss compared with their urban counterparts. This contrasts with studies from India and Zimbabwe, possibly due to differences in socioeconomic conditions or other regional factors affecting pregnancy outcomes [52, 55]. Studies suggest that environmental aspects of rural areas have been associated with longer survival times to pregnancy loss [73]. The natural environment in rural areas may contribute to better overall well‐being and reduced stress, potentially leading to longer survival times for women experiencing pregnancy loss.

In this study, significant regional variations in the risk of pregnancy loss were observed even after adjusting for individual‐level and contextual covariates. Areas such as northeastern Kenya, southern Mozambique, and northeastern and western Tanzania exhibited lower frailty. These persistent spatial patterns suggest the presence of unmeasured regional factors influencing pregnancy outcomes (Figure 2). Possible explanations for these disparities include inequitable access to quality maternal healthcare services, delays in seeking care due to cultural or gender norms, and socioeconomic disadvantages that limit women′s ability to access timely and adequate reproductive care. Additionally, environmental stressors such as food insecurity, infectious disease burden, and poor sanitation may exacerbate the risk of pregnancy complications in these regions. The spatial frailty map thus provides valuable insight into where targeted maternal health interventions, infrastructure investment, and resource allocation could be most effective in reducing pregnancy loss across East Africa.

This study highlights key opportunities to reduce pregnancy loss in East Africa by addressing both individual‐ and region‐level risk factors. Targeted interventions are particularly needed in high‐risk areas identified through the spatial frailty analysis. Persistent regional disparities suggest that structural barriers, limited access to quality maternal healthcare, and context‐specific social or environmental factors contribute to elevated risk. These findings provide actionable guidance for policymakers to prioritize resource allocation, improve maternal health services, and implement context‐sensitive strategies aimed at reducing pregnancy loss across the region.

### 4.1. Strength and Limitation of the Study

This study benefits from nationally representative data, enhancing reliability and generalizability. The Bayesian spatial survival model enabled robust estimation by accounting for both observed covariates and unmeasured spatial heterogeneity. However, several limitations should be noted. Although DHS guidelines were followed, the timing of the recent survey varies across countries, which may affect comparability. Other limitations include reliance on retrospective self‐reports, which may introduce recall bias, the use of predefined regional boundaries (MAUP), differences in survey years across countries, and the inability to account for recurrent pregnancy loss events due to the DHS data structure, which only captures the most recent pregnancy within the 5‐year recall period. Despite these limitations, the study provides valuable insights into the individual‐ and region‐level determinants of pregnancy loss in East Africa.

## 5. Conclusions

Pregnancy loss remains a major public health concern in East Africa, characterized by marked spatial disparities that persist even after accounting for known risk factors. High‐risk groups include older women, those with limited education, women without partners, and urban residents. Targeted interventions are needed in high‐risk regions to improve healthcare access, strengthen prenatal care, and expand community education. Further research should explore the underlying contextual and structural factors driving regional disparities to guide more effective, evidence‐based interventions.

**Nomenclature**
AF: acceleration factor
AFT: accelerated failure time
BF: Bayes factor
CrI: credible interval
DIC: Deviance information criteria
ENAP: Every Newborn Action Plan
LPML: log pseudo marginal likelihood
MCMC: Markov‐chain Monte‐Carlo
SBR: stillbirth rate
WAIC: Watanabe–Akaike information criterion

## Author Contributions

**S.K.A.** contributed to conceptualization, methodology, data curation, software, data analysis, and manuscript drafting and revision. **M.A.A.** contributed to methodology, supervision, validation, and manuscript revision. **T.A.A.** contributed to methodology, supervision, validation, and critical revision of the manuscript.

## Funding

No funding was received for this manuscript.

## Consent

The authors have nothing to report.

## Conflicts of Interest

The authors declare no conflicts of interest.

## Supporting Information

Additional supporting information can be found online in the Supporting Information section.

## Supporting information


**Supporting Information 1.** Annex S1 includes detailed statistical modeling procedures, covering spatial analysis, standard survival analysis, and the accelerated failure time (AFT) model with frailty. It also describes the Bayesian spatial survival modeling approach, including spatial frailty specification, prior distributions, and variable selection procedures.

**Supporting Information 2.** Annex S2 presents model diagnostics, comparison, and convergence assessment results. Nonparametric survival analysis methods are also included. The Cox–Snell residual plots indicate an overall good model fit.

## Data Availability

The data utilized in this study were accessed from the Demographic and Health Surveys (DHS) Program database following a formal registration process. Interested researchers can obtain the data by registering and submitting a request via the DHS Program′s website at (https://dhsprogram.com).

## References

[1] Kuppusamy P. , Prusty R. K. , Chaaithanya I. K. , Gajbhiye R. K. , and Sachdeva G. , Pregnancy Outcomes Among Indian Women: Increased Prevalence of Miscarriage and Stillbirth During 2015–2021, BMC Pregnancy and Childbirth. (2023) 23, no. 1, 10.1186/s12884-023-05470-3, 36890450. PMC9992916 36890450

[2] Da Silva F. T. , Gonik B. , McMillan M. , Keech C. , Dellicour S. , Bhange S. , Tila M. , Harper D. M. , Woods C. , Kawai A. T. , Kochhar S. , Munoz F. M. , and Brighton Collaboration Stillbirth Working Group , Stillbirth: Case Definition and Guidelines for Data Collection, Analysis, and Presentation of Maternal Immunization Safety Data, Vaccine. (2016) 34, no. 49, 6057–6068, 10.1016/j.vaccine.2016.03.044, 27431422. 27431422 PMC5139804

[3] Kowaleski J. , State Definitions and Reporting Requirements for Live Births, Fetal Deaths, and Induced Terminations of Pregnancy: US Department of Health and Human Services, Public Health Service, 1997, National Center for Health Statistics.

[4] Allanson E. R. , Tunçalp Ö. , Gardosi J. , Pattinson R. C. , Francis A. , Vogel J. P. , Erwich J. , Flenady V. J. , Frøen J. F. , Neilson J. , Quach A. , Chou D. , Mathai M. , Say L. , and Gülmezoglu A. M. , The WHO Application Of ICD-10 To Deaths During The Perinatal period (ICD‐PM): Results From Pilot Database Testing In South Africa And United Kingdom, Journal of Obstetrics & Gynaecology. (2016) 123, no. 12, 2019–2028, 10.1111/1471-0528.14244. 27527122

[5] Cuenca D. , Pregnancy Loss: Consequences for Mental Health, Frontiers in Global Women′s Health. (2023) 3, 1032212, 10.3389/fgwh.2022.1032212, 36817872. PMC9937061 36817872

[6] Wilcox A. J. , Weinberg C. R. , Wehmann R. E. , Armstrong E. G. , Canfield R. E. , and Nisula B. C. , Measuring Early Pregnancy Loss: Laboratory and Field Methods, Fertility and Sterility. (1985) 44, no. 3, 366–374, 10.1016/S0015-0282(16)48862-X, 4029425. 4029425

[7] Quenby S. , Gallos I. D. , Dhillon-Smith R. K. , Podesek M. , Stephenson M. D. , Fisher J. , Brosens J. J. , Brewin J. , Ramhorst R. , Lucas E. S. , McCoy R. C. , Anderson R. , Daher S. , Regan L. , al-Memar M. , Bourne T. , MacIntyre D. A. , Rai R. , Christiansen O. B. , Sugiura-Ogasawara M. , Odendaal J. , Devall A. J. , Bennett P. R. , Petrou S. , and Coomarasamy A. , Miscarriage Matters: the Epidemiological, Physical, Psychological, and Economic Costs of Early Pregnancy Loss, Lancet. (2021) 397, no. 10285, 1658–1667, 10.1016/S0140-6736(21)00682-6, 33915094. 33915094

[8] UNICEF , A Neglected Tragedy 2020, 2025, https://data.unicef.org/resources/a-neglected-tragedy-stillbirth-estimates-report/.

[9] World Health Organization , Protect the Progress: Rise, Refocus and Recover: 2020 Progress Report on the Every Woman Every Child Global Strategy for Women′s, Children′s and Adolescents’ Health 2016-2030, 2020, https://www.who.int/publications/i/item/9789240011991.

[10] Endawkie A. and Tsega Y. , Pregnancy Loss and Its Predictors Among Ever-Pregnant Women in Sub-Saharan Africa: Multilevel Mixed Effect Negative Binomial Regression, PLOS Global Public Health. (2025) 5, no. 4, e0004316, 10.1371/journal.pgph.0004316, 40193521. 40193521 PMC11975090

[11] Jackson T. and Watkins E. , Early Pregnancy Loss, Journal of the American Academy of Physician Associates. (2021) 34, no. 3, 22–27, 10.1097/01.JAA.0000733216.66078.ac. 33528169

[12] American College of Obstetricians and Gynecologists’ Committee on Practice Bulletins-Gynecology , ACOG Practice Bulletin No. 200: Early Pregnancy Loss, Obstetrics and Gynecology. (2018) 132, no. 5, e197–e207, 10.1097/AOG.0000000000002899. 30157093

[13] Altijani N. , Carson C. , Choudhury S. S. , Rani A. , Sarma U. C. , Knight M. , and Nair M. , Stillbirth Among Women in Nine States in India: Rate and Risk Factors in Study of 886, 505 Women From the Annual Health Survey, BMJ Open. (2018) 8, no. 11, e022583, 10.1136/bmjopen-2018-022583, 30413502. PMC6231551 30413502

[14] Aminu M. , Unkels R. , Mdegela M. , Utz B. , Adaji S. , and Van Den Broek N. , Causes of and Factors Associated With Stillbirth in low‐ and Middle-Income Countries: A Systematic Literature Review, BJOG: An International Journal of Obstetrics & Gynaecology. (2014) 121, no. s4, 141–153, 10.1111/1471-0528.12995, 25236649. 25236649

[15] Neill S. , Management of Early Pregnancy Loss, Journal of the American Medical Association. (2023) 329, no. 16, 1399–1400, 10.1001/jama.2023.0933. 37027174

[16] Kasa G. A. , Woldemariam A. Y. , Adella A. , and Alemu B. , The Factors Associated With Stillbirths Among Sub-Saharan African Deliveries: A Systematic Review and Meta-Analysis, BMC Pregnancy and Childbirth. (2023) 23, no. 1, 10.1186/s12884-023-06148-6, 38049743. PMC10696713 38049743

[17] Berhie K. A. and Gebresilassie H. G. , Logistic Regression Analysis on the Determinants of Stillbirth in Ethiopia, Maternal Health, Neonatology and Perinatology. (2016) 2, no. 1, 10.1186/s40748-016-0038-5, 27660718. PMC5025573 27660718

[18] Lema G. , Mremi A. , Amsi P. , Pyuza J. J. , Alloyce J. P. , Mchome B. , and Mlay P. , Placental Pathology and Maternal Factors Associated With Stillbirth: An Institutional Based Case-Control Study in Northern Tanzania, PLoS One. (2020) 15, no. 12, e0243455, 10.1371/journal.pone.0243455, 33382728. 33382728 PMC7775101

[19] Worede D. T. and Dagnew G. W. , Determinants of stillbirth in Felege-Hiwot Comprehensive Specialized Referral Hospital, North-west, Ethiopia, 2019, BMC Research Notes. (2019) 12, no. 1, 10.1186/s13104-019-4621-5, 31521188. PMC6744638 31521188

[20] Moodley Y. , Eilerts H. , Herbst K. , and Tanser F. , Pregnancy Loss and Postpartum Mortality in a Sub-Saharan African Setting, preprint, medRxiv, 2021 10.1101/2021.03.18.21253872.

[21] deMontigny F. , Verdon C. , Meunier S. , Gervais C. , and Coté I. , Protective and Risk Factors for Women′s Mental Health After a Spontaneous Abortion, Revista Latino-Americana De Enfermagem. (2020) 28, e3350, 10.1590/1518-8345.3382.3350, 32901768. 32901768 PMC7478879

[22] Klier C. M. , Geller P. A. , and Ritsher J. B. , Affective Disorders in the Aftermath of Miscarriage: A Comprehensive Review, Archives of Women′s Mental Health. (2002) 5, no. 4, 129–149, 10.1007/s00737-002-0146-2, 12510205. 12510205

[23] Ganatra B. , Gerdts C. , Rossier C. , Johnson B. R. , Tunçalp Ö. , Assifi A. , Sedgh G. , Singh S. , Bankole A. , Popinchalk A. , Bearak J. , Kang Z. , and Alkema L. , Global, Regional, and Subregional Classification of Abortions by Safety, 2010–14: Estimates From a Bayesian Hierarchical Model, Lancet. (2017) 390, no. 10110, 2372–2381, 10.1016/S0140-6736(17)31794-4, 28964589. 28964589 PMC5711001

[24] UNICEF , Stillbirth, 2025, https://data.unicef.org/topic/child-survival/stillbirths/.

[25] Lawn J. E. , Blencowe H. , Waiswa P. , Amouzou A. , Mathers C. , Hogan D. , and Draper E. , Stillbirths: Rates, Risk Factors and Potential for Progress Towards 2030, Lancet. (2016) 387, no. 10018, 587–603. 26794078 10.1016/S0140-6736(15)00837-5

[26] Tesema G. A. , Mekonnen T. H. , and Teshale A. B. , Spatial Distribution and Determinants of Abortion Among Reproductive Age Women in Ethiopia, Evidence From Ethiopian Demographic and Health Survey 2016 Data: Spatial and Mixed-Effect Analysis, PloS One. (2020) 15, no. 6, e0235382, 10.1371/journal.pone.0235382, 32598398. 32598398 PMC7323954

[27] Tesema G. A. , Gezie L. D. , and Nigatu S. G. , Spatial Distribution of Stillbirth and Associated Factors in Ethiopia: A Spatial and Multilevel Analysis, BMJ Open. (2020) 10, no. 10, e034562, 10.1136/bmjopen-2019-034562, 33115888. PMC7594361 33115888

[28] Assefa N. , Berhane Y. , Worku A. , and Tsui A. , The Hazard of Pregnancy Loss and Stillbirth Among Women in Kersa, East Ethiopia: A Follow Up Study, Sexual & Reproductive Healthcare. (2012) 3, no. 3, 107–112, 10.1016/j.srhc.2012.06.002, 22980735. 22980735

[29] Ibrahim J. G. , Chen M.-H. , and Sinha D. , Bayesian Survival Analysis, 2001, Springer Science & Business Media, 10.1007/978-1-4757-3447-8.

[30] Zhou H. and Hanson T. , Mitra R. and Muller P. , Bayesian Spatial Survival Models, Biostatistics, 2015, Springer, 215–246, 10.1007/978-3-319-19518-6_11.

[31] Bartoš F. , Aust F. , and Haaf J. M. , Informed Bayesian Survival Analysis, BMC Medical Research Methodology. (2022) 22, no. 1, 10.1186/s12874-022-01676-9, 36088281. PMC9464410 36088281

[32] DHS , The DHS Program, 2023, https://dhsprogram.com/countries/country-list.cfm.

[33] WHO , WHO Recommendations on Antenatal Care for a Positive Pregnancy Experience, 2016, World Health Organization. 28079998

[34] Van Buuren S. and Groothuis-Oudshoorn K. , mice: Multivariate Imputation by Chained Equations inR, Journal of Statistical Software. (2011) 45, no. 3, 1–67, 10.18637/jss.v045.i03.

[35] Wang S. , Zhang J. , and Lawson A. B. , A Bayesian Normal Mixture Accelerated Failure Time Spatial Model and Its Application to Prostate Cancer, Statistical Methods in Medical Research. (2016) 25, no. 2, 793–806, 10.1177/0962280212466189, 23117407. 23117407

[36] Keiding N. , Andersen P. K. , and Klein J. P. , The Role of Frailty Models and Accelerated Failure Time Models in Describing Heterogeneity due to Omitted Covariates, Statistics in Medicine. (1998) 16, no. 2, 215–224, 10.1002/(SICI)1097-0258(19970130)16:2<215::AID-SIM481>3.0.CO;2-J. 9004393

[37] Collett D. , Modelling Survival Data in Medical Research, 2023, Chapman and Hall/CRC, 10.1201/9781003282525.

[38] Zhou H. , Hanson T. , and Zhang J. , Generalized Accelerated Failure Time Spatial Frailty Model for Arbitrarily Censored Data, Lifetime Data Analysis. (2017) 23, no. 3, 495–515, 10.1007/s10985-016-9361-4, 26993982. 26993982 PMC5352560

[39] Zhou H. , Hanson T. , and Zhang J. , spBayesSurv: Fitting Bayesian Spatial Survival Models UsingR, Journal of Statistical Software. (2020) 92, no. 9, 1–33, 10.18637/jss.v092.i09.

[40] Zhou H. , Hanson T. , Jara A. , and Zhang J. , Modelling County Level Breast Cancer Survival Data Using a Covariate-Adjusted Frailty Proportional Hazards Model, Annals of Applied Statistics. (2015) 9, no. 1, 43–68, 10.1214/14-AOAS793, 26236420. 26236420 PMC4520441

[41] Müller P. , Quintana F. A. , Jara A. , and Hanson T. , Bayesian Nonparametric Data Analysis, 2015, Springer, 10.1007/978-3-319-18968-0.

[42] Lavine M. L. and Hodges J. S. , On Rigorous Specification of ICAR Models, American Statistician.(2012) 66, no. 1, 42–49, 10.1080/00031305.2012.654746.

[43] Malsiner-Walli G. and Wagner H. , Comparing Spike and Slab Priors for Bayesian Variable Selection, preprint, arXiv, 2018 10.48550/arXiv.1812.07259.

[44] Cox D. R. and Snell E. J. , A General Definition of Residuals, Journal of the Royal Statistical Society: Series B. (1968) 30, no. 2, 248–265, 10.1111/j.2517-6161.1968.tb00724.x.

[45] Andersen A. M. N. , Wohlfahrt J. , Christens P. , Olsen J. , and Melbye M. , Maternal Age and Fetal Loss: Population Based Register Linkage Study, British Medical Journal. (2000) 320, no. 7251, 1708–1712, 10.1136/bmj.320.7251.1708, 10864550. 10864550 PMC27416

[46] Frederiksen L. E. , Ernst A. , Brix N. , Braskhøj Lauridsen L. L. , Roos L. , Ramlau-Hansen C. H. , and Ekelund C. K. , Risk of Adverse Pregnancy Outcomes at Advanced Maternal Age, Obstetrics & Gynecology. (2018) 131, no. 3, 457–463, 10.1097/AOG.0000000000002504. 29420406

[47] Nguyen B. T. , Chang E. J. , and Bendikson K. A. , Advanced Paternal Age and the Risk of Spontaneous Abortion: an Analysis of the Combined 2011–2013 and 2013–2015 National Survey of Family Growth, American Journal of Obstetrics and Gynecology.(2019) 221, no. 5, 476.e1–476.e7, 10.1016/j.ajog.2019.05.028, 31128112. 31128112

[48] Caserta D. , Ralli E. , Matteucci E. , Bordi G. , Soave I. , Marci R. , and Moscarini F. , The Influence of Socio-Demographic Factors on Miscarriage Incidence Among Italian and Immigrant Women: A Critical Analysis From Italy, Journal of Immigrant and Minority Health. (2015) 17, no. 3, 843–851, 10.1007/s10903-014-0005-z, 24627173. 24627173

[49] Feodor Nilsson S. , Andersen P. , Strandberg-Larsen K. , and Nybo Andersen A. M. , Risk Factors for Miscarriage From a Prevention Perspective: A Nationwide Follow-Up Study, BJOG: An International Journal of Obstetrics & Gynaecology. (2014) 121, no. 11, 1375–1385, 10.1111/1471-0528.12694, 24548778. 24548778

[50] Balayla J. , Azoulay L. , and Abenhaim H. A. , Maternal Marital Status and the Risk of Stillbirth and Infant Death: a Population-Based Cohort Study on 40 Million Births in the United States, Women′s Health Issues. (2011) 21, no. 5, 361–365, 10.1016/j.whi.2011.04.001, 21689945. 21689945

[51] Tesema G. A. , Wolde M. , Tamirat K. S. , Worku M. G. , Fente B. M. , Tsega S. S. , Tadesse A. , and Teshale A. B. , Factors Associated With Short Birth Interval Among Reproductive-Age Women in East Africa, Women′s Health. (2023) 19, 17455057231209879, 10.1177/17455057231209879, 37955253. PMC10644753 37955253

[52] Fletcher R. , Forbes F. , Dadi A. F. , Kassa G. M. , Regan C. , Galle A. , Beyene A. , Liackman R. , and Temmerman M. , Effect of Male Partners′ Involvement and Support on Reproductive, Maternal and Child Health and Well-Being in East Africa: A Scoping Review, Health Science Reports. (2024) 7, no. 8, e2269, 10.1002/hsr2.2269, 39086507. 39086507 PMC11286546

[53] Shapiro G. D. , Bushnik T. , Wilkins R. , Kramer M. S. , Kaufman J. S. , Sheppard A. J. , and Yang S. , Adverse Birth Outcomes in Relation to Maternal Marital and Cohabitation Status in Canada, Annals of Epidemiology. (2018) 28, no. 8, 503–509-e11, 10.1016/j.annepidem.2018.05.001. 29937402

[54] Cheng E. R. , Rifas-Shiman S. L. , Perkins M. E. , Rich-Edwards J. W. , Gillman M. W. , Wright R. , and Taveras E. M. , The Influence of Antenatal Partner Support on Pregnancy Outcomes, Journal of Women′s Health. (2016) 25, no. 7, 672–679, 10.1089/jwh.2015.5462, 26828630. PMC4985003 26828630

[55] Fatema K. and Lariscy J. T. , Mass Media Exposure and Maternal Healthcare Utilization in South Asia, SSM-Population Health. (2020) 11, 100614, 10.1016/j.ssmph.2020.100614, 32596437. 32596437 PMC7306581

[56] Sserwanja Q. , Mutisya L. M. , and Musaba M. W. , Exposure to Different Types of Mass Media and Timing of Antenatal Care Initiation: Insights From the 2016 Uganda Demographic and Health Survey, BMC Women′s Health. (2022) 22, no. 1, 10.1186/s12905-022-01594-4, 35012537. PMC8751065 35012537

[57] Tesfa G. A. , Getnet A. , and Seboka B. T. , Unmet Maternal Health Information Needs and Mass Media Exposure for Maternal Health Among Women in the Gedeo zone, South Ethiopia, South Ethiopia. Frontiers in Public Health. (2025) 13, 1497606, 10.3389/fpubh.2025.1497606, 40771254. 40771254 PMC12325304

[58] Corchero-Falcón M. D. R. , Gómez-Salgado J. , García-Iglesias J. J. , Camacho-Vega J. C. , Fagundo-Rivera J. , and Carrasco-González A. M. , Risk Factors for Working Pregnant Women and Potential Adverse Consequences of Exposure: A Systematic Review, International Journal of Public Health. (2023) 68, 1605655, 10.3389/ijph.2023.1605655. 36874222 PMC9977819

[59] Mozurkewich E. L. , Luke B. , Avni M. , and Wolf F. M. , Working Conditions and Adverse Pregnancy Outcome: A Meta-Analysis, Obstetrics & Gynecology. (2000) 95, no. 4, 623–635, 10.1097/00006250-200004000-00029, 10725502. 10725502

[60] Yihune Teshale M. , Bante A. , Gedefaw Belete A. , Crutzen R. , Spigt M. , and Stutterheim S. E. , Barriers and Facilitators to Maternal Healthcare in East Africa: a Systematic Review and Qualitative Synthesis of Perspectives From Women, Their Families, Healthcare Providers, and Key Stakeholders, BMC Pregnancy and Childbirth. (2025) 25, no. 1, 10.1186/s12884-025-07225-8, 39901111. PMC11792318 39901111

[61] Di Nallo A. and Köksal S. , Job Loss During Pregnancy and the Risk of Miscarriage and Stillbirth, Human Reproduction. (2023) 38, no. 11, 2259–2266, 10.1093/humrep/dead183, 37758648. 37758648 PMC10628490

[62] Weitzman A. , The effects of Women′s Education on Maternal Health: Evidence From Peru, Social Science & Medicine. (2017) 180, 1–9, 10.1016/j.socscimed.2017.03.004, 28301806. 28301806 PMC5423409

[63] Nicholls-Dempsey L. , Badeghiesh A. , Baghlaf H. , and Dahan M. H. , How Does High Socioeconomic Status Affect Maternal and Neonatal Pregnancy outcomes? A population-Based Study Among American Women, European Journal of Obstetrics & Gynecology and Reproductive Biology: X. (2023) 20, 100248, 10.1016/j.eurox.2023.100248. 37876770 10.1016/j.eurox.2023.100248 PMC10590715

[64] Kramer M. S. , Séguin L. , Lydon J. , and Goulet L. , Socio-Economic Disparities in Pregnancy Outcome: Why Do the Poor Fare So Poorly?, Paediatric and Perinatal Epidemiology. (2000) 14, no. 3, 194–210, 10.1046/j.1365-3016.2000.00266.x, 10949211. 10949211

[65] Tahira T. and Fatima D. , Literacy on Pregnancy Complications: Causal Factors and Prevention, Advances in Healthcare Research. (2024) 2, no. 2, 116–129, 10.60079/ahr.v2i2.374.

[66] Samuel K. G. , Kandala N.-B. , Ryan B. L. , and Thind A. , Predictors of Pregnancy Loss Among Urban and Rural Women Aged 15 to 49 Years in Pakistan, BMC Public Health. (2025) 25, no. 1, 10.1186/s12889-025-22165-w, 40065258. PMC11895280 40065258

[67] Regassa L. D. , Tola A. , Daraje G. , and Dheresa M. , Trends and Determinants of Pregnancy Loss in Eastern Ethiopia From 2008 to 2019: Analysis of Health and Demographic Surveillance Data, BMC Pregnancy and Childbirth. (2022) 22, no. 1, 10.1186/s12884-022-04994-4, 36045340. PMC9429487 36045340

[68] Dahiru T. and Aliyu A. A. , Stillbirth in Nigeria: Rates and Risk Factors Based on 2013 Nigeria DHS, Open Access Library Journal. (2016) 3, no. 8, 1–12, 10.4236/oalib.1102747.

[69] Mwenebanda E. , Machado A. , Patel A. I. , Nyondo-Mipando A. L. , and Chiumia I. K. , Factors Influencing Antenatal Care Attendance in the Eight Contact Era Policy: A Case of Selected Maternal Health Service Facilities in Blantyre, Malawi, BMC Pregnancy and Childbirth. (2024) 24, no. 1, 10.1186/s12884-024-06895-0, 39462321. PMC11514860 39462321

[70] Gomindes A. R. , Bhakthavalsalan R. , Sharma U. , Johnston S. L. , Johnston S. , and Naushad A. , Prevalence of High-Risk Pregnancy Among Pregnant Women Attending Antenatal Care Camps in Primary Health Centres in Kinaye and Vantamuri and Their Sub-Centres, Cureus. (2022) 14, no. 7, 10.7759/cureus.27355. PMC9417325 36046327

[71] Mare K. U. , Andargie G. G. , Moloro A. H. , Mohammed A. A. , Mohammed O. A. , Wengoro B. F. , Lahole B. K. , Hadaro T. S. , Leyto S. M. , Mamo P. O. , Hedato A. H. , Seifu B. L. , Wondmeneh T. G. , Ebrahim O. A. , and Sabo K. G. , Late Initiation of Antenatal Care Visit Amid Implementation of New Antenatal Care Model in Sub-Saharan African Countries: A Multilevel Analysis of Multination Population Survey Data, PloS One. (2025) 20, no. 1, e0316671, 10.1371/journal.pone.0316671, 39888887. 39888887 PMC11785278

[72] Ahrens K. A. , Rossen L. M. , and Branum A. M. , Pregnancy Loss History at First Parity and Selected Adverse Pregnancy Outcomes, Annals of Epidemiology. (2016) 26, no. 7, 474–481.e9, 10.1016/j.annepidem.2016.04.011, 27262817. 27262817 PMC6626662

[73] Kumari U. , Sharma R. K. , Keshari J. , and Sinha A. , Environmental Exposure: Effect on Maternal Morbidity and Mortality and Neonatal Health, Cureus. (2023) 15, no. 5, e38548, 10.7759/cureus.38548. 37273345 PMC10239284

