# Supplementary material for: Geospatial Variation and Determinants of Time to Pregnancy Loss Among Reproductive‐Aged Women in East Africa: Bayesian Spatial Frailty Model
Source: Biomed Res Int. 2026 Jun 19;2026:4637028. doi: 10.1155/bmri/4637028 (PMC13280569; doi:10.1155/bmri/4637028)
Supplement: Supplementary file 1 — Supporting Information 1. Annex S1 includes detailed statistical modeling procedures, covering spatial analysis, standard survival analysis, and the accelerated failure time (AFT) model with frailty. It also describes the Bayesian spatial survival modeling approach, including spatial frailty specification, prior distributions, and variable selection procedures. [file BMRI-2026-4637028-s002.pdf]

Spatial analysis

Spatial data analysis was conducted using R version 4.4.1 to examine geographic patterns of pregnancy loss among women in East Africa. Coordinate points in shape file format obtained from DHS, along with region shape files obtained from GADM website (1) were used for each country. Pregnancy loss proportions were calculated for each survey cluster. Clusters with missing or zero latitude and longitude data were cleaned before merging.

Spatial weights were constructed using the queen contiguity method, which identifies neighboring regions based on shared borders.

Survival Analysis

To determine the duration until the occurrence of pregnancy loss, the Kaplan-Meier (K-M) method was employed (2), which is a non-parametric approach that enables the estimation of survival probabilities over time. We then implemented the log-rank test (3) to evaluate disparities in survival times among different categorical variables. In a proportional hazards (PH) model, a key consideration is the assumption that the survival curves for different groups or strata should have hazard functions that remain proportional over time (4). In order to verify the proportional hazard model's assumption we employed the global Schoenfeld residuals test (5).

Proportional Hazard model with Frailty

The term frailty model is used to denote a survival regression model that incorporates random effect distinct classes (e.g. regions). A Cox model with mixed effects can be formulated as (6)

$$h_i(\mathrm{t}) = h_0(t)\exp(x'\beta + \alpha_j)$$

where α_j denotes the random effect associated with the jth region. Rabe-Hesketh and Skrondal use the term 'shared frailty' to denote the exponential of the random effect: exp^{α_j} (7). The shared frailty term has a multiplicative effect on the baseline hazard function:

$$h_i(\mathrm{t}) = h_0(t)exp^{\alpha_j}\exp(x'\beta)$$ or we can also write it as

$$\eta_i = x_i\beta + v_i,$$ where v_i is a random effect, termed "frailty" (4).

In a proportional hazards (PH) model, a key consideration is the assumption that the survival curves for different groups or strata should have hazard functions that remain proportional over time. When the proportional hazards assumption is violated, results from a PH model will be

difficult to generalize to situations with different follow-up periods and challenging to interpret. The accelerated failure time (AFT) model offers an alternative approach for analyzing time-to-event data, remaining applicable even when hazards are not proportional and often used as an important alternative to the PH model in practice (8).

Accelerated Failure Time model with frailty

Although the Cox (1972) PH models are highly popular, AFT models have two key advantages: (a) they offer a straightforward interpretation of regression parameters through a log-linear formulation, and (b) their parameter estimates are more robust to omitted covariates in contrast to PH models (9, 10). AFT models assume that covariates accelerate or decelerate the time to an event by a constant factor.

Bayesian spatial survival analysis

We analyzed the time to pregnancy loss using Bayesian spatial survival models, employing both semiparametric and parametric approaches. To ensure the convergence of the Markov Chain Monte Carlo (MCMC) simulations, we implemented a burn-in period of 6000 iterations. After the burn-in, we retained 8000 iterations for posterior analysis, with every 20th iteration saved to reduce autocorrelation between samples. The model fitting was conducted using the spBayesSurv package in R, which efficiently handles spatial survival data within a Bayesian framework (11). We evaluated Bayesian semi parametric and parametric spatial models with proportional odds (PO), proportional hazards (PH), and accelerated failure time (AFT) frameworks, incorporating various baseline distributions. To capture unobserved factors, we included frailty terms in these models to account for spatial dependencies.

Statistical models

Bayesian modeling approach for spatial survival analysis

The Bayesian analysis approach stands as an independent method, distinct from classical analysis approaches (12). In Bayesian analysis, parameters are estimated through the examination of the posterior distribution. This distribution is derived from a combination of prior information and the likelihood of the observed data (13-15).

Spatial dependence often arises among survival outcomes. Such spatial dependence is often due to region specific similarities are typically not measurable. When survival data are spatially

correlated, it is often of scientific interest to investigate possible spatial dependence in survival outcomes after adjusting for known subject-specific covariate effects. Frailty models are one approach to incorporate spatial dependences in the models (16).

Spatial Frailty Modeling

The spatial structure can be incorporated into the AFT model by adding a random effect, resulting in the AFT spatial model (17);

$$y_{ij} = log^{(t_{ij})} = x_{ij}{}^{T}\ \beta + \mathrm{vi} + \epsilon_{ij}$$

The AFT model with frailty has survival and density functions (18);

$$S_{xij}(t) = S_0\left(\exp\left(x_{ij}^{\mathsf{T}}\beta + v_{ij}t\right)\right), \quad f_{xij}(t) = \exp\left(x_{ij}^{\mathsf{T}}\beta + v_{ij}\right)f_0\left(\exp\left(x_{ij}^{\mathsf{T}}\beta + v_{ij}t\right)\right)$$

Where, $\beta = \left(\beta_1, \dots, \beta_p\right)^{\mathsf{T}}$ i s a vector of regression coefficients, v_i is an unobserved frailty associated with s_i, and " S_0(t)" is the baseline survival with density " f_0(t)" corresponding to " X_{ij}=0" and " v_i = 0

In the case of spatial survival data, we can extend the frailty model by including a spatial effect as (16).

$$\eta_i = x_i\beta + \gamma_i, \quad \gamma_i = v_i + w_i,$$

where the frailty term γi incorporates the effects of both heterogeneity (via the non-spatial frailty vi) and spatial dependence (through the spatial frailty wi).

In studies involving areal data, the entire study region (East Africa in our case) R is typically divided into a finite number of areas (countries region), denoted as B1, B2, ..., BG. Within each of these regions, a common frailty is assumed for all subjects. This approach acknowledges that individuals within the same region may share certain unobserved characteristics that affect the outcome of interest, such as time to pregnancy loss in this case., i.e.

$$\eta_i = x_i\beta + \gamma_{g_i}, \quad \gamma_j = v_j + w_j, \quad j = 1, \dots, G.$$

In this context, the non-spatial frailty v_j for each region is usually assumed to follow a normal distribution with a mean of zero and a variance of σ^2.

For the spatial frailty term w_j,, two main approaches have been used (16). These are the Gaussian Random Field (GRF) model and an intrinsic conditionally autoregressive (ICAR) model (16). In this study we utilized the ICAR model for modeling spatial dependency.

Conditionally autoregressive (ICAR) model

In modeling time to pregnancy loss across nine East African countries, we incorporated region-level frailty using a conditionally autoregressive (ICAR) model. This approach utilizes spatial relationships based on whether regions share borders. Specifically, the spatial structure is defined by an adjacency matrix A where $a_{ij} = 1$ if areas B_i and B_j share a nontrivial border (i.e., a connected curve in R2 that is more than one point) and $a_{ij} = 0$ otherwise; set $a_{ii} = 0$.

The 169×169 adjacency matrix (that we obtained from regions of 9 countries) A captures these relationships for our dataset. In the ICAR model, regions that share borders are assumed to have similar frailty values. The ICAR prior is established through a set of conditional distributions with (16, 19).

$$w_j|(w_i: i = j) \sim N\left(\overline{w_j}, \frac{\theta^2}{a_j^+}\right), \quad j = 1, \ldots, G.$$

Denoted $w \sim ICAR\left(\frac{1}{\theta^2}\right)$, Where a_{j+} is the number of neighbors of region B_j , $\bar{w}_j = \frac{1}{a_{j+}} \sum_{i:a_{ij}=1}, w_i$ is the sample mean of the a_{j+}values of the neighboring regional unit frailties, and $\frac{\theta^2}{a_{j+}}$ is the conditional variance (16).

Likelihood function in Bayesian spatial survival analysis:

Suppose we observe n independent vectors of (t_i, θ), where t_i, is time to the event and δ_i is indicator variable telling us whether t_i, is censored or not, i.e, t_i, = 0 for censored observation (δ_i= 0) and t_i = 1 for uncensored observation (δ_i = 1). The spatial survival frailty likelihood function of the set of unknown parameters θ in the presence of right censoring is given a (W_L,θ, β, v) is given (11)

$$L(w_L, \theta, \beta, v) = \sum_{i=1}^{m} \sum_{j=1}^{n_i} \left(S_{x_{ij}}(a_{ij}) - S_{x_{ij}}(b_{ij})\right) I^{a_{ij}<b_{ij}} f_{x_{ij}}(a_{ij}) I^{a_{ij}=b_{ij}}$$

Where; W_L: Spatial frailty term for each area, representing unobserved spatial effects. θ: Parameter in the covariance matrix for the spatial frailty term, β: Coefficients for the fixed effects in the model, v: non-spatial frailty term for each area (18).

Prior specification

Given that spatial frailty models had not been previously employed to study the hazard of pregnancy loss, and there was no prior information available regarding the values of the spatial random effects or substantive covariates in the presence of these spatial frailties, we used vague priors. Consequently, the regression coefficients were assumed to follow a normal distribution

$$\beta \sim N(0,1000)$$

For the baseline hazard function scale parameter (S0), we assigned Bayesian nonparametric vague priors to the shape (α) and scale (θ) parameters of the Two-Parameter Burr Type XII transformed Bernstein polynomial (TBPL) distribution ($S0(\cdot) \mid \alpha, \theta \sim TBP_L\ (\alpha, S_\theta\ (\cdot))$), as follows (19, 20), Therefore, the baseline hazard prior specification can be written as;

$$S0(\cdot) \mid \alpha, \theta \sim TBP_L\ (\alpha, S_\theta\ (\cdot)),\ \alpha \sim \Gamma\ (0.01\ ,0.01) \quad \text{and}\ \theta \sim N_2\ (0,1000)$$

Prior specification for frailty terms

In the case of the spatial frailty terms V_i, vague priors was assigned to the precision parameter τ in the Intrinsic Conditional Autoregressive (ICAR) model with a gamma distribution (21).

$$(v1, \ldots, vm)^T|\tau \sim \text{ICAR}\ (\tau^2), \tau^{-2} \sim \Gamma(0.01\ ,0.01)$$

These non-informative priors allow the data to strongly influence the posterior distribution.

Variable selection

Spike-and-slab prior (22) with the Bernoulli distribution was used for variable selection implemented through the spBayesSurv R package (11).

Model diagnostics and comparison

We diagnosed the model using Cox and Snell residual (23). We assessed the model using the deviance information criterion (DIC) (24), the log pseudo marginal likelihood (25) and the Watanabe-Akaike information criterion (WAIC) (26). A better model was the one with a smaller

DIC and WAIC value and a larger LPML (27). Bayes factors were also utilized to compare the performance of semiparametric models with underlying parametric models (18).

Assessment of convergence

To ensure the reliability of our Markov Chain Monte Carlo (MCMC) analysis results, we verified that the chain has reached its stationary distribution. We also assessed both the Bulk Effective Sample Size and Tail Effective Sample Size were adequate (28). Additionally, we inspected trace plots for each chain to confirm they exhibit good mixing and examine density plots to assess their smoothness. Furthermore we assessed the Autocorrelation to evaluate the independence of samples (29, 30).

References

1. GADM. 2024 [Available from: https://gadm.org/.
2. Kaplan EL, Meier P. Nonparametric estimation from incomplete observations. Journal of the American statistical association. 1958;53(282):457-81.
3. Mantel N. Evaluation of survival data and two new rank order statistics arising in its consideration. Cancer Chemother Rep. 1966;50(3):163-70.
4. Kleinbaum DG, Klein M. Survival analysis a self-learning text: Springer; 1996.
5. Schoenfeld D. Partial residuals for the proportional hazards regression model. Biometrika. 1982;69(1):239-41.
6. Austin PC. A tutorial on multilevel survival analysis: methods, models and applications. International Statistical Review. 2017;85(2):185-203.
7. Rabe-Hesketh S, Skrondal A. Multilevel and Longitudinal Modeling Using Stata, Volumes I and II: Multilevel and Longitudinal Modeling Using Stata, Volume II... Counts, and Survival. Stata Press; 2012.
8. Wang S, Zhang J, Lawson AB. A Bayesian normal mixture accelerated failure time spatial model and its application to prostate cancer. Statistical methods in medical research. 2016;25(2):793-806.

9. Keiding N, Andersen PK, Klein JP. The role of frailty models and accelerated failure time models in describing heterogeneity due to omitted covariates. Statistics in medicine. 1997;16(2):215-24.
10. Hougaard P. Fundamentals of survival data. Biometrics. 1999;55(1):13-22.
11. Zhou H, Hanson T, Zhang J. spBayesSurv: Fitting Bayesian spatial survival models using R. arXiv preprint arXiv:170504584. 2017.
12. Gelman A, Carlin JB, Stern HS, Rubin DB. Bayesian data analysis: Chapman and Hall/CRC; 1995.
13. Bürkner P-C. brms: An R package for Bayesian multilevel models using Stan. Journal of statistical software. 2017;80:1-28.
14. Bartoš F, Aust F, Haaf JM. Informed Bayesian survival analysis. BMC Medical Research Methodology. 2022;22(1):238.
15. Ibrahim JG, Chen M-H, Sinha D, Ibrahim J, Chen M. Bayesian survival analysis: Springer; 2001.
16. Zhou H, Hanson T. Bayesian spatial survival models. Nonparametric Bayesian Inference in Biostatistics. 2015:215-46.
17. Collett D. Modelling survival data in medical research: Chapman and Hall/CRC; 2023.
18. Zhou H, Hanson T, Zhang J. Generalized accelerated failure time spatial frailty model for arbitrarily censored data. Lifetime data analysis. 2017;23:495-515.
19. Zhou H, Hanson T, Jara A, Zhang J. Modelling county level breast cancer survival data using a covariate-adjusted frailty proportional hazards model. The annals of applied statistics. 2015;9(1):43.
20. Müller P, Quintana FA, Jara A, Hanson T. Bayesian nonparametric data analysis: Springer; 2015.
21. Lavine ML, Hodges JS. On rigorous specification of ICAR models. The American Statistician. 2012;66(1):42-9.
22. Malsiner-Walli G, Wagner H. Comparing spike and slab priors for Bayesian variable selection. arXiv preprint arXiv:181207259. 2018.
23. Cox DR, Snell EJ. A general definition of residuals. Journal of the Royal Statistical Society: Series B (Methodological). 1968;30(2):248-65.

24. Spiegelhalter DJ, Best NG, Carlin BP, Van Der Linde A. Bayesian measures of model complexity and fit. Journal of the royal statistical society: Series b (statistical methodology). 2002;64(4):583-639.
25. Geisser S, Eddy WF. A predictive approach to model selection. Journal of the American Statistical Association. 1979;74(365):153-60.
26. Watanabe S, Opper M. Asymptotic equivalence of Bayes cross validation and widely applicable information criterion in singular learning theory. Journal of machine learning research. 2010;11(12).
27. Zhou H, Hanson T. A unified framework for fitting Bayesian semiparametric models to arbitrarily censored survival data, including spatially referenced data. Journal of the American Statistical Association. 2018;113(522):571-81.
28. Vehtari A, Gelman A, Simpson D, Carpenter B, Bürkner P-C. Rank-normalization, folding, and localization: An improved Rˆ for assessing convergence of MCMC (with discussion). Bayesian analysis. 2021;16(2):667-718.
29. Gelman A, Rubin DB. Inference from iterative simulation using multiple sequences. Statistical science. 1992;7(4):457-72.
30. Brooks SP, Gelman A. General methods for monitoring convergence of iterative simulations. Journal of computational and graphical statistics. 1998;7(4):434-55.
