# Supplementary material for: Geospatial Variation and Determinants of Time to Pregnancy Loss Among Reproductive‐Aged Women in East Africa: Bayesian Spatial Frailty Model
Source: Biomed Res Int. 2026 Jun 19;2026:4637028. doi: 10.1155/bmri/4637028 (PMC13280569; doi:10.1155/bmri/4637028)
Supplement: Supplementary file 2 — Supporting Information 2. Annex S2 presents model diagnostics, comparison, and convergence assessment results. Nonparametric survival analysis methods are also included. The Cox–Snell residual plots indicate an overall good model fit. [file BMRI-2026-4637028-s001.pdf]

## Survival Analysis

### Survival analysis using non-parametric methods

The survival time of East African women experiencing pregnancy loss, as represented by the Kaplan-Meier survival estimate, showed a survival curve that declines at a fairly steady rate. The median survival time of pregnancy loss was not defined **(Figure 1).**

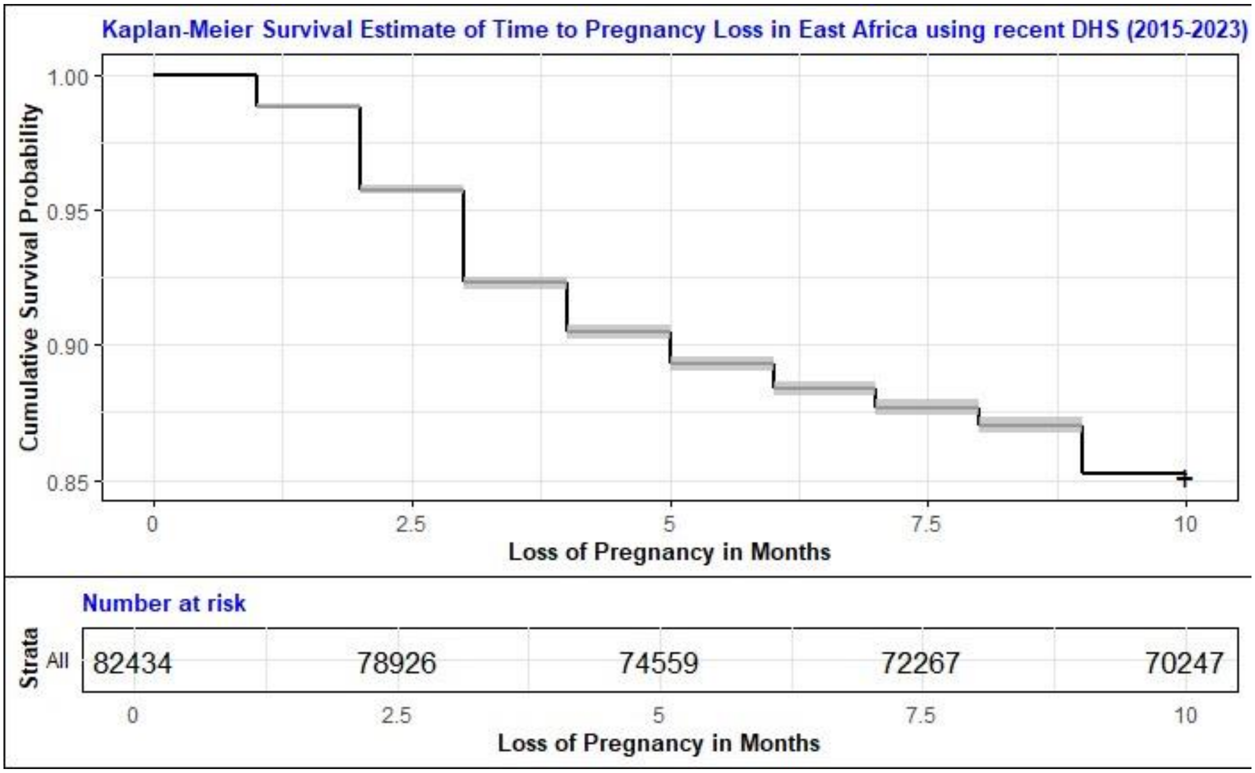


***Figure 1: Kaplan Meier survival estimate of pregnancy loss in East Africa using recent DHS (2015-2023)***

The Kaplan Meir survival analysis reveals that pregnant women who lives in urban have lower survival probabilities compared to rural counterparts. Additionally, women who underwent cesarean sections experienced lower survival rates compared to those who had spontaneous vaginal deliveries. Furthermore, younger maternal and paternal ages are associated with higher survival estimates compared to older ages **(Figure 2).**

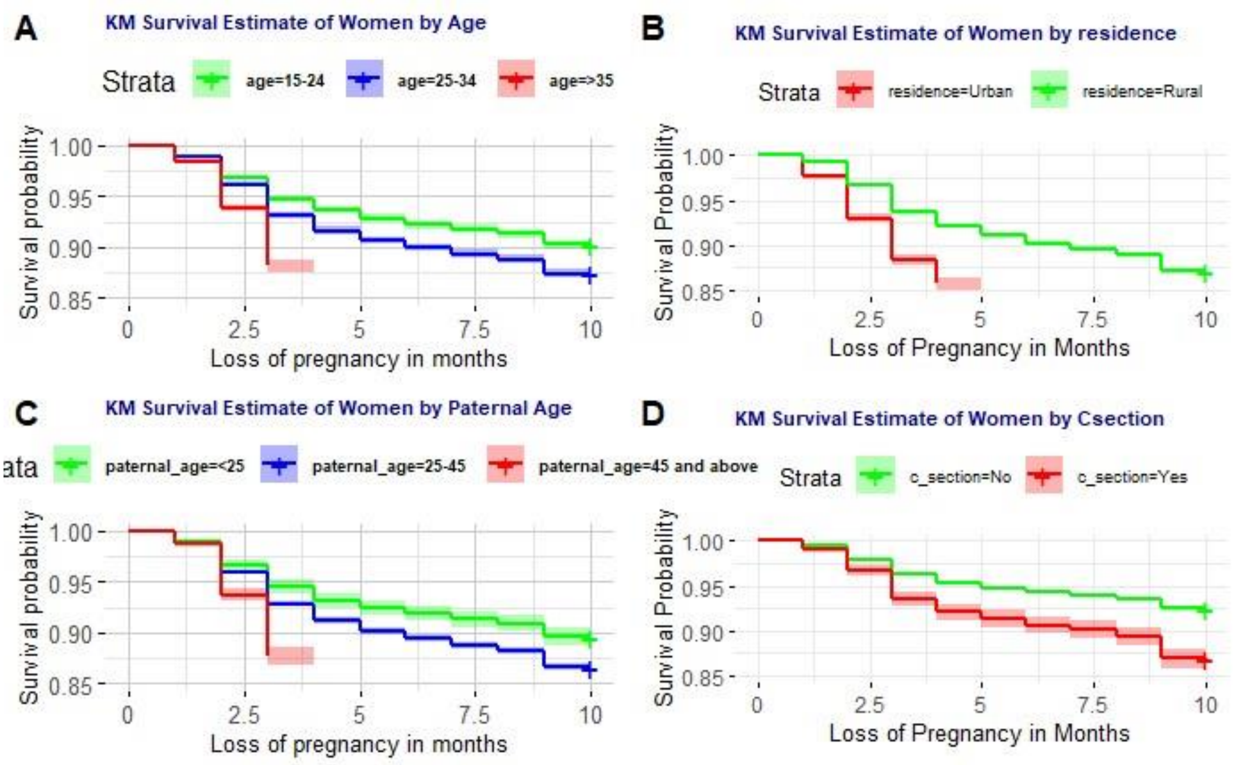


***Figure 2: Survival estimates of pregnant women by women's Age (Fig. A), survival estimates of pregnant women by residence (Fig. B), and survival estimates of women by paternal age (Fig. C), survival estimates of women by Cesarean section (Fig. D)***

To evaluate the differences in survival experiences across various categorical variables the log-rank test was conducted. The results, detailed in Table 1, reveal significant disparities in survival across several factors. There are statistically significant differences in survival based on maternal age, country, media exposure, women's occupation, health insurance, residence, parity, cesarean section, ANC visit, women's educational level, husband's occupation, contraceptive use, wealth status, and previous loss.

Table 1: Log-rank test examining the factors associated with time to pregnancy loss in East Africa using recent DHS (2015-2023)

| **Variable** | **Log rank test** | **P-value** | **Variable** | **Log rank test** | **P value** | **Variable** | **Log rank test** | **P value** |
|---|---|---|---|---|---|---|---|---|
| Maternal age | 2124.48 | 0.000 | Country | 3299.79 | 0.000 | Media exposure | 696.426 | 0.000 |

| | | | | | | | | |
|---|---|---|---|---|---|---|---|---|
| Women occupation | 248.14 | 0.000 | Smoking habit | 2.61 | 0.000 | Health insurance | 45.08 | 0.000 |
| Residence | 615.58 | 0.000 | Parity | 13367.07 | 0.000 | cesarean section | 205.93 | 0.000 |
| Marital status | 0.80 | 0.371 | ANC visit | 112.78 | 0.000 | Birth interval | 3.10 | 0.078 |
| Women's educational level | 300.73 | 0.000 | Husband occupation | 72.00 | 0.000 | Husband educational status | 206.57 | 0.000 |
| Contraceptive | 604.92 | 0.000 | Wealth status | 596.35 | 0.000 | Previous loss | 744.01 | 0.000 |

**Test of proportionality hazard assumption**

The Schoenfeld residual test indicates that many factors did not remain constant over time, and the global test confirms a violation of the proportional hazards (PH) assumption as shown in Table 52below. As a result, we fitted an accelerated failure time (AFT) model.

Table 2: Schoenfeld residuals test for checking proportional hazard assumption for the time to pregnancy loss and its associated factors in East Africa using recent DHS (2015-2023)

| **Variable** | **Chi-square** | **P value** | **Variable** | **Chi-square** | **P value** |
|---|---|---|---|---|---|
| Maternal age | 49.67 | 0.000 | Insurance | 71.76 | 0.000 |
| Women occupation | 4.1 | 0.043 | Cesarean section | 0.12 | 0.733 |
| Residence | 82.9 | 0.000 | Parity | 405.65 | 0.000 |
| Marital status | 10.85 | 0.001 | ANC visit | 14.53 | 0.000 |
| Women's educational level | 248.28 | 0.000 | Media exposure | 32.1 | 0.000 |
| **Global test** | 839.82 | 0.000 | | | |

**Bayesian spatial AFT Model**

After we selected AFT model for survival analysis to model time to pregnancy loss, we incorporated region-level frailty using a conditionally autoregressive (ICAR) model across nine East African countries. Spike and slab variable selection was then conducted to determine the final covariates that were included in multivariable regression.

**Model diagnostics and comparison**

The semiparametric model with ICAR frailty with a lognormal distribution performed better. This model had the smallest Deviance Information Criterion (DIC), the largest Log Pseudo Marginal Likelihood (LPML), and the smallest Watanabe-Akaike Information Criterion (WAIC). To assess whether the parametric model outperformed the log-normal parametric model, we compared it using Bayes Factors. As shown in Table 3, with a Bayes Factor (5209.56) the results indicate that the ICAR model, a semiparametric approach, was superior to the log-normal parametric model

Table *3*: Model comparisons with and without frailty for different baseline distribution

| **Distribution** | **No frailty** | | | **ICAR Frailty** | | |
|---|---|---|---|---|---|---|
| | **DIC** | **LPML** | **WAIC** | **DIC** | **LPML** | **WAIC** |
| **Lognormal** | 150,417.5 | -73,211.76 | 150,521.5 | 113,905.1 | -56,952.89 | 113,905.8 |
| **Weibull** | 169,841.1 | -78,253.70 | 169,901.2 | 125,921.6 | -62253.70 | 125,934.2 |
| **Log logistic** | 178,459.9 | -84,496.92 | 178,541.9 | 128,989.3 | -64496.92 | 128,993.9 |
| ***DIC*** *- Deviance Information Criterion;* ***LPML-*** *Log Pseudo Marginal Likelihood;* ***WAIC*** *-Watanabe-Akaike Information Criterion* | | | | | | |

To assess the goodness of fit of the model We generated Cox-Snell plots using 10 posterior residuals. The plot indicates a generally good fit of the survival model to the data, as most of the points follow the 45-degree line closely **(see Figure 3).**

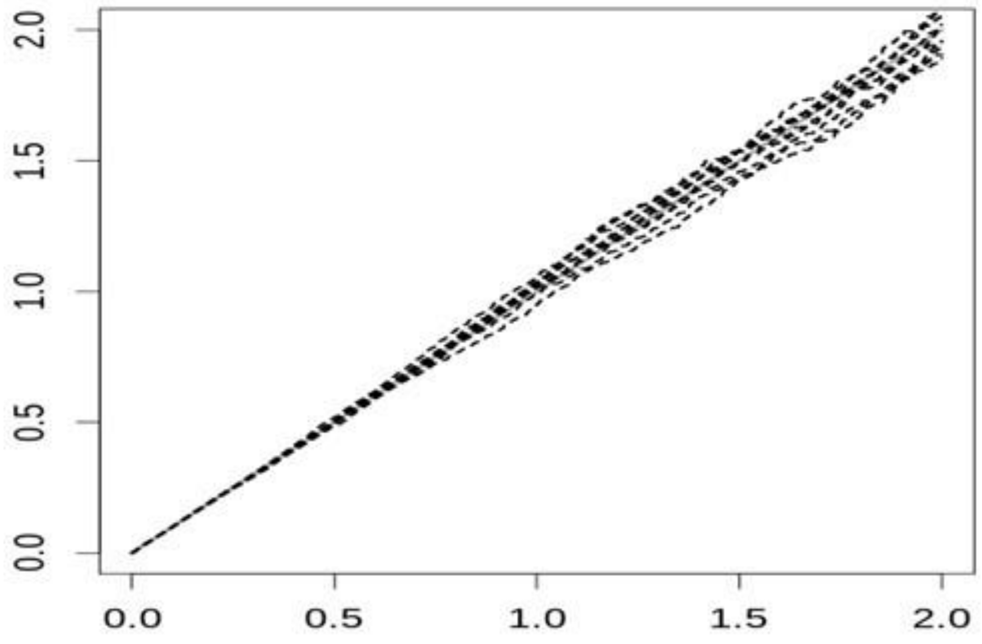


***Figure 3: Cox Snell residual plot of the lognormal distribution with ICAR frailty***

**Assessment of convergence**

Model convergence was confirmed with the Bulk Effective Sample Size and Tail Effective Sample Size for all parameters were above 2500 and 3100, respectively. Additionally, the trace plots showed good mixing, and the density plots were smooth (see Figure 4).

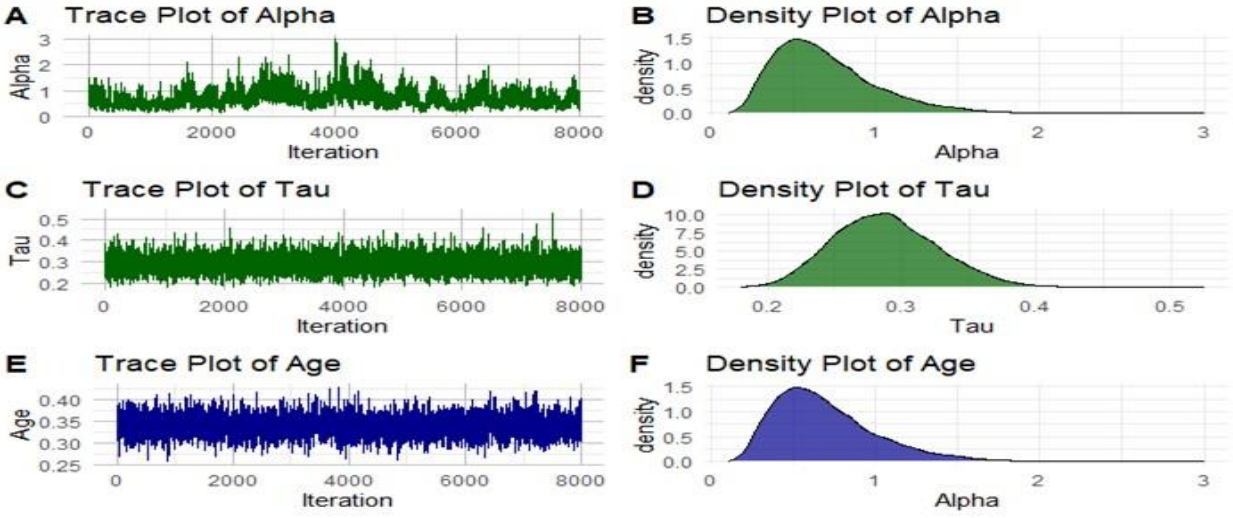


**Figure 4: Trace and density plot of some of the covariates and parameters**
